# Field trial of an intervention targeting social images of healthy eating and vegetable consumption among military conscripts

**DOI:** 10.1177/13591053251396771

**Published:** 2026-01-11

**Authors:** Marja Ilona Kinnunen, Matthias Burkard Aulbach, Piia Jallinoja, Ari Haukkala, Nelli Hankonen, Clarissa Bingham, Antti Uutela, Pilvikki Absetz

**Affiliations:** 1 Finnish Musculoskeletal Association, Helsinki, Finland; 2 National Institute for Health and Welfare, Helsinki, Finland; 3 Paris-Lodron-University of Salzburg, Austria; 4 Tampere University, Finland; 5 University of Helsinki, Finland; 6 Laurea University of Applied Sciences, Helsinki, Finland

**Keywords:** prototypes, vegetables, intervention, health promotion, eating behavior

## Abstract

Young people’s eating behavior is influenced by their prototypes of typical healthy and unhealthy peer eaters. This study evaluates the effects of a poster and cartoon strip campaign to promote positive images of vegetable eating peers and vegetable consumption among conscripted men in a quasi-experimental study design. Samples of young Finnish men reported vegetable consumption frequency at the start of compulsory military service (control arm *n* = 848, intervention arm *n* = 959) and at 6 months of service (control *n* = 424, intervention *n* = 484). Half of the participants reported their perceptions of Vegetable chooser or Vegetable abstainer prototypes, respectively, at baseline and follow-up (control *n* = 239, intervention *n* = 234). Three out of six prototypes became less positive toward vegetable consumption at follow-up. However, these trends were less pronounced in the intervention than control group for prototypes and vegetable consumption.

## Introduction

Fruits and vegetables are available for most people but in many countries their consumption does not meet the WHO’s recommendation of 400 g per individual per day (Hall et al., 2009). Adult Finnish men consume far less fruit and vegetables than recommended (Helakorpi et al., 2010; Valsta et al., 2018) and young men entering compulsory military service make no exception (Bingham et al., 2011). Furthermore, socioeconomic health differences (Elgar et al., 2015) are partially explained by differences in health behaviors, vegetable consumption being one of them (Laaksonen et al., 2008). A socioeconomic gradient in fruit and vegetable consumption has been found among Finnish adults (Konttinen et al., 2013) and also conscripted men: Men with upper secondary or higher education entering military service consume more vegetables than those with comprehensive school or vocational education only (Absetz et al., 2010). Thus, it is of interest to evaluate whether health promotion efforts equally influence socioeconomic groups (Hankonen et al., 2009) or whether they in fact enlarge the gap. Every eligible man in Finland is liable to military service and about 80% in each age cohort completes the service (at the time of data collection; The Finnish Defence Forces, 2010). Therefore, military service provides an excellent opportunity to examine, and influence eating and related psychosocial variables among young men of all backgrounds.

The schedules and meal patterns in garrison (i.e. the military facility housing soldiers) favor functionality (Hoikkala et al., 2009). As 98% of Finnish soldiers are men, masculine food preferences prevail as exemplified by not favoring vegetables (Ruby and Heine, 2011). Masculinity is emphasized both in the training and the prevailing culture, and healthy eating, often with attributes such as “light,” do not fit well with it (Hoikkala et al., 2009).

Health promotion strategies targeting individuals’ vegetable consumption often assume individuals’ changes in health behaviors are based on explicit rational decisions resulting in intentions, as postulated by the Theory of Planned Behavior (Ajzen, 2002) or the Health Action Process Approach (HAPA-model, Schwarzer, 2008). The Theory of Planned Behavior explains behavior through the formation of intentions, which are shaped by attitudes, perceived norms, and perceived control. The Health Action Process Approach builds on this by specifying additional determinants of intention, such as risk perception and outcome expectancies, and by emphasizing self-regulatory processes that help translate intentions into actual behavior. Both models therefore conceptualize health behavior as the outcome of reasoned, planful processes. However, it has become increasingly clear that impulsive psychological processes influence health behaviors in a way that can counteract rational decision making (Hofmann et al., 2008) and that these processes should be specifically targeted in health behavior interventions (Marteau et al., 2012).

The Prototype Willingness model (Gerrard et al., 2008; Gibbons et al., 2020) does not assume rational deliberation as a prerequisite for action. Instead, it emphasizes an additional, socially cued pathway in which prototypes—images of a typical peer engaging in a behavior—shape behavioral willingness and can influence behavior even in the absence of strong intentions. These prototypes contribute to behavior independently from intentions and are particularly important in adolescents and young adults whose behaviors are often guided by spontaneous and socially influenced processes: adolescents hold clear images of typical (un)healthy eaters and those influence their own eating behavior (Dohnke et al., 2015; Gerrits et al., 2009). Critically for this study, vegetable abstainer and vegetable chooser prototypes are associated with fruit and vegetable consumption among young men (Jallinoja et al., 2011). Especially among the youth (Gerrard et al., 2008), influencing positive or negative perceptions of individuals who consume vegetables may be one useful intervention strategy (Todd et al., 2016), but so far it has been rarely tested in the area of healthy eating.

The objective of this study was to evaluate an intervention among conscripted men to promote *vegetable consumption* via the automatic processing pathway, specifically by *promoting attachment of positive characteristics to the prototype of a vegetable eating peer* and thereby making it more appealing than a vegetable abstainer prototype. Similar processes are also well documented in food advertising and media portrayals, where images of eaters are strategically constructed to make foods appear more desirable or socially valued. Such media representations demonstrate that social images can shape food-related attitudes and behaviors, and prototype-based interventions can be seen as applying similar mechanisms to promote healthier social images (Jelicich and Braun, 2023). The specific aims of the present study were to examine (1) intervention effectiveness in changing influencing positive or negative perceptions of vegetable consumers (vegetable chooser and vegetable abstainer), (2) intervention effectiveness in changing vegetable consumption, (3) whether changes in intentions to eat fruit and vegetables are related to changes in prototypes and changes in vegetable consumption, and (4) whether socioeconomic status measured by education moderate intervention effects on both prototypes and vegetable consumption.

## Methods

### Study setting and design

At the garrison, conscripts are served three healthy meals daily (breakfast, lunch, and dinner). Also a voluntary evening snack is served at the garrison’s canteen, and conscripts may acquire food and drink from other food parlors in- and outside the garrison. On all meals during the service, men pick up their meals from a buffet table where various salads and fresh vegetables are always available. The present study is part of a two-phase intervention study (Bingham et al., 2011, 2012; Jallinoja et al., 2011) conducted in two garrisons in Finland between 2007 and 2009. Data collected in 2007 served as control data. The study protocol was approved by the ethics committee of the hospital district of Helsinki and Uusimaa, Finland.

### Intervention

Similar to other health behaviors, regional differences in vegetable consumption in Finland are significant (Helakorpi et al., 2010). Therefore, the same two garrisons were used throughout the three study years with two batches of new conscripts entering in each of them annually. Data collected in 2008 served as a control group for the intervention that took place in 2009. The 2009 intervention consisted of *a campaign to promote a positive, socially acceptable, masculine image of vegetable eaters (*i.e. *prototypes)*. The campaign included two different posters to hang on the walls of service units, canteens and barracks and three different triangle-shaped comic adverts displayed on the tables in barracks and canteens. These materials were on view twice for all conscripts between the measurements, for a period of 2 weeks each time. The intervention materials included drawn pictures and texts that humorously combined masculine garrison culture to suggest that vegetables are an important part in the arsenal of every soldier (see Kinnunen, 2016: 75–79 for images of the posters in Finnish language). From each garrison, people in charge of the visibility of the intervention campaign reported the starting and end point of the material displays.

### Participants

From each of the two brigades, two service units took part in the study. For the control group, the number of men in the units were 1087 and for the Intervention group 1038. The target sample size was based on the names enlisted for the study units and was evaluated to be sufficient to reliably detect effects of a meaningful size. Of those enlisted, 82% (848) in the control and 88% (959) in the intervention condition filled in the questionnaire at baseline. The follow-up questionnaire at 6 months was filled in by 584 (retention 69%) in the control and 631 (retention 66%) in the intervention group. All men included in the study approved and signed the informed consent form that contained information that the study was about attitudes to eating but no details about the intervention’s specifics and aims. Dropout was either due to interruption of service, being ill during measurements, military transfer, or refusal to attend the study. At follow-up, dropout was also due to being on encampment or on leave.

### Measures

The same measures, described in detail elsewhere (reference not shown), were used at baseline and 6 months later.

*Vegetable consumption* was assessed as part of a food frequency questionnaire. The question “On how many days during the last seven days did you consume fresh vegetables and salads” had eight response possibilities ranging from *“on 0 days”* to *“on 7 days.”*

*Education* was measured with one question: “What is the highest education you have completed?” Forty-four percent of the participants reported having completed upper secondary school (1% reported having completed college-level or higher training, 42% had vocational education, and 12% had completed secondary school). In the analyses two educational categories were used (1 = secondary or vocational school and 2 = upper-secondary or college-level/higher education).

*Intention* was measured with a single item, in line with several studies using the HAPA model (Schwarzer, 2008). *“What kind of intentions do you have during the following weeks and months? I intend to eat plenty of fruit and vegetables”*. The fixed answering alternatives ranged from *“I definitely do not intend”* (1) to *“I definitely intend to”* (7).

#### Prototypes

The question stem: “We will ask you to evaluate a typical conscript, who behaves in a way described below” was followed by one of the two descriptions:

(A) “A typical conscript who tries to choose fruits and vegetables at every meal.”
(B) “A typical conscript who tries to avoid vegetables and fruits.”

The statements were followed by 17 adjective pairs, such as “easy going-straight laced,” “popular-unpopular,” “unreliable-reliable,” “masculine-feminine.” Participants were asked to mark the point on the five-point semantic differential between the adjectives that most accurately represented their opinion. Three main dimensions were identified in all four prototypes, with appropriate reversions applied where necessary: *Self-regulation* (reliable, grown-up, strict, responsible, and intelligent), *Social status in peer group* (easy-going, popular, masculine, and good-company) and *Good-Looking* (muscular, trendy, good-looking, thin, and physically fit). Factors and their associations to respective eating style have been published elsewhere (reference not shown).

To reduce measurement burden in prototypes, half of the participants filled in questionnaires involving “Vegetable Chooser” (statement A above)/“Vegetable Abstainer” (statement B above) prototypes (Control arm: Baseline *n* = 431, Follow-up *n* = 424, and intervention arm: Baseline *n* = 484, Follow-up *n* = 321). Each participant within this half answered either questions about “Vegetable Abstainer” or “Vegetable Chooser.” The other half answered questions on “Fat Chooser”/“Fat Abstainer prototypes.” As the intervention was targeting vegetable prototypes, only results concerning them are reported in this paper.

### Statistical analyses

To examine the intervention effect on prototypes mixed within-between ANOVAs were run for all six prototypes separately, with the study arm as the between- and time as the within-subjects factor. Equivalent analyses investigated the effect on vegetable consumption. Change scores for intention, vegetable consumption and relevant prototype factors were calculated by subtracting the baseline value from the follow-up value. Pearson correlations between the change scores were used in the analyses. All analyses were conducted with SPSS 22.0.

## Results

The means for baseline and follow-up in Vegetable Chooser and Vegetable Abstainer prototypes in both groups can be seen in Table 1. In both groups, Vegetable Chooser prototypes were rated more highly on *Self-regulation* and *Appearance* at baseline than Vegetable Abstainer prototypes (*p* < 0.001).

**Table 1.** Means (M), standard deviations (SD), and *F*-values for vegetable chooser and vegetable avoider prototypes at baseline and 6 months measures, in the intervention group and the control group.

	Control group				Intervention group					
	*N* = 209				*N* = 234					
	Baseline		6 months		Baseline		6 months		Within subjects *F*	
	*M*	SD	*M*	SD	*M*	SD	*M*	SD	Main effect	Interaction
Vegetable chooser										
Self-regulation	3.47	0.53	3.29	0.52	3.38	0.51	3.34	0.53	5.67*	5.70*
Social standing in peer group	3.18	0.48	3.17	0.58	3.19	0.53	3.19	0.56	0.01	0.00
Appearance	3.38	0.45	3.30	0.53	3.31	0.49	3.31	0.48	1.73	2.61
Vegetable avoider										
Self-regulation	2.68	0.59	2.87	0.53	2.78	0.54	2.79	0.55	12.58**	8.53**
Social standing in peer group	3.12	0.5	3.10	0.53	3.11	0.43	3.10	0.51	0.49	0.06
Appearance	2.7	0.55	2.85	0.54	2.81	0.54	2.84	0.56	8.16**	4.25**

* p < 0.05. **p < 0.001.

### Intervention effectiveness in influencing perceptions of vegetable consumers

#### Changes on vegetable chooser prototype

Ratings on the *Self-regulation* factor declined over the 6 months in service across groups [*F*(1,441) = 15.85, *p* < 0.001]. The time X condition interaction term was significant [*F*(1,441) = 5.70, *p* = 0.017] indicating that the size of the negative change occurred more strongly in the control than the intervention group. There were no significant effects in the factors *Social standing in peer group* and *Appearance*; they were similar at baseline and after 6 months in both control and intervention groups.

#### Changes on vegetable abstainer prototype

The ratings on the *Self-regulation* factor increased during the 6 months in service [*F*(1,441) = 12.58, *p* < 0.001], but the significant interaction with study arm suggested that the negative change occurred more strongly in the control group than in the intervention group [*F*(1,441) = 8.56, *p* = 0.004]. Similarly, the *Appearance* factor was rated higher after 6 months in service [*F*(1,441) = 8.16, *p* = 0.004]. This was mainly due to changes in the control group’s ratings as suggested by the significant interaction term [*F*(1,441) = 4.26, *p* = 0.04]. There were no significant changes or interactions in the *Social standing in peer group* factor: it was rated as highly at baseline and after 6 months of service in both control and intervention groups.

### Intervention effectiveness in changing vegetable consumption

As the intervention reached all men in the research units (not just those who provided evaluations of Vegetable Chooser and Abstainer prototypes), the vegetable consumption of all available participants was analyzed. The means (and standard deviations) for the vegetable consumption at baseline and at 6 months are in the intervention group 3.04 and 2.90 (2.17, 2.00), and in the control group 3.33 and 2.98 (2.15, 1.95). The within subjects analysis showed that vegetable consumption declined significantly during the 6 months in service [*F*(1,906) = 17.75, *p* < 0.001]. A significant interaction between time and study arm [*F*(1,906) = 4.76, *p* = 0.029] indicated that the decrease was significantly smaller in the intervention group.

### Changes in intentions to eat fruit and vegetables are related to changes in prototypes and changes in vegetable consumption

The change score analyses indicated that the intention to eat fruit and vegetables declined during the 6 months in military service [*F*(1,900) = 31.69, *p* < 0.001] and that the intervention did not influence intention (time × study arm interaction [*F*(1,900) = 3.23, *p* < 0.073]). The means and standard deviations for the change scores in intention and vegetable consumption are presented in Table 2, the correlations with other change score in Table 3. The correlations between the change scores in the intentions to eat plenty of fruit and vegetables were not significant with other change scores in the control group. However, in the intervention group, increase in intentions was associated with a more positive change score in Vegetable chooser Self-regulation prototype (*r* = 0.20, *p* = 0.017) and more positive change in vegetable consumption (*r* = 0.11, *p* = 0.002).

**Table 2.** Means (M) and standard deviations (SD) for the change scores (T2–T1) in intention to eat fruit and vegetables (FV), in vegetable consumption, and in FV prototypes in the intervention group and the control group.

Condition	Change in FV intention	Change in FV consumption	Change in FV chooser self-regulation	Change in FV avoider self-regulation	Change in FV avoider appearance
Control *M* (SD*)*	−0.39 (1.66)	−0.45 (2.07)	−0.18 (0.60)	0.19 (0.66)	0.15 (0.65)
Intervention *M* (SD)	−0.21 (1.49)	−0.14 (2.20)	−0.04 (0.58)	0.13 (0.59)	0.03 (0.65)

**Table 3.** Correlations between fruits and vegetables (FV) intention change, prototype changes, change in intention to eat fruit and vegetables, and change in vegetable consumption.

Change variables (T2-T1)	Change in FV intention		Change in FV consumption	
	Control	Intervention	Control	Intervention
Change in FV intention	1	1	0.01	0.11*
Change in CV chooser self-regulation	0.12	0.20**	−0.01	−0.05
Change in FV avoider self-regulation	−0.08	−0.10	0.07	−0.04
Change in FV avoider appearance	−0.10	−0.01	−0.04	0.05

* p = 0.017. **p = 0.002.

### Educational differences in levels and changes in prototypes, intention, and vegetable consumption

Higher education was associated with higher ratings of *Self-regulation* [*F*(1,431) = 10.32, *p* < 0.001] and *Social standing* factors of the Vegetable Chooser prototype [*F*(1,431) = 3.92, *p* = 0.048] at baseline, but there were no significant interactions between intervention arm and education.

Higher education was also associated with lower ratings of *Self-regulation* [*F*(1,431) = 14,10, *p* < 0.001] and *Appearance* factors [*F*(1,428) = 5.83, *p* < 0.001] of the Vegetable Abstainer prototype at baseline. Significant interactions between study arm, time, and education [*F*(1,428) = 5,70, *p* = 0.017] in the *Self-regulation* and *Appearance* factors [*F*(1,428) = 4,98, *p* = 0.026] of the Vegetable Abstainer prototype indicate that the less negative prototype development in the intervention group (compared to the control group) occurred mainly among those with higher education.

**Figure 1.**
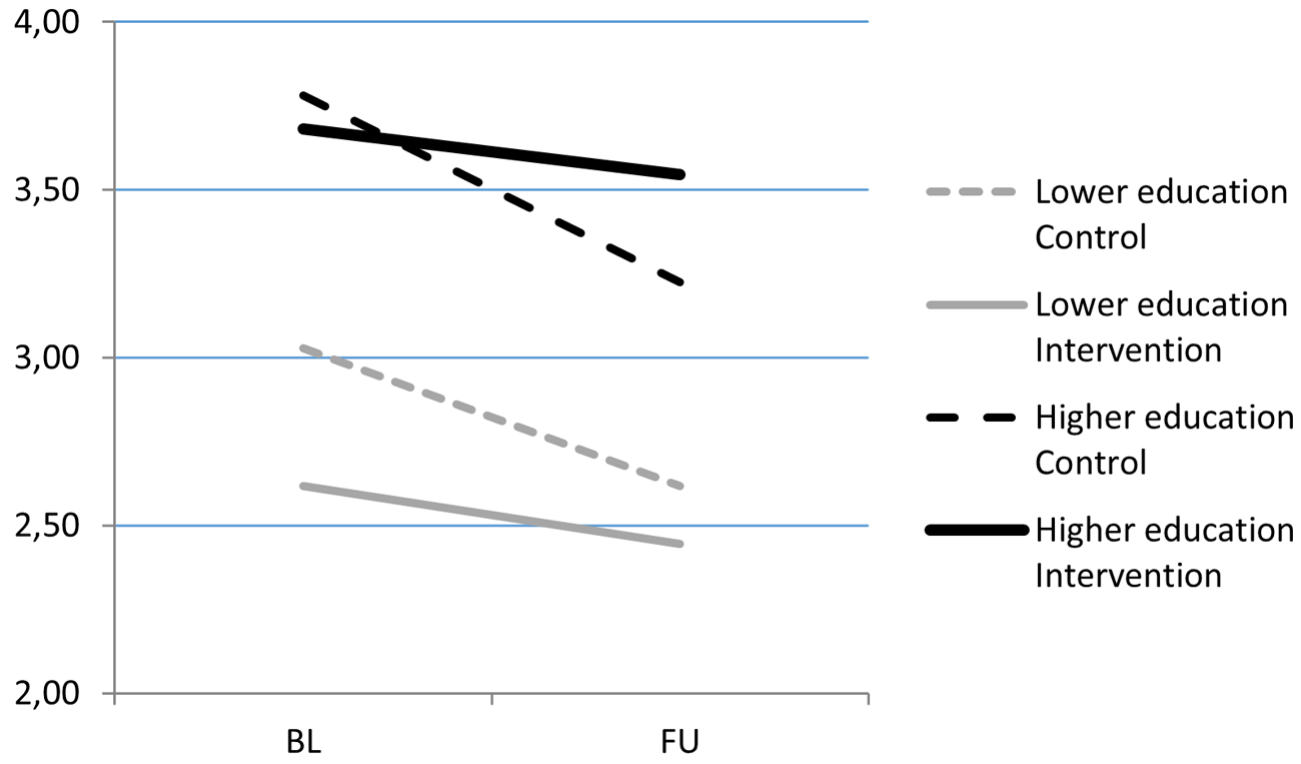
Fruit and vegetable consumption at baseline (BL) and at 6 months (FU) by education, in the intervention group and the control groups.

At baseline, participants with higher education reported significantly higher vegetable consumption [*F*(1,1780) = 157.31, *p* < 0.001]. Vegetable consumption declined to a similar degree during the 6 months in both educational groups (interaction between vegetable consumption, study arm, and education [*F*(1,887) = 0.381, *p* = 0.537]; Figure 1).

Higher education was associated with higher intentions to eat fruit and vegetables at baseline [*F*(1,1772) = 72.41, *p* < 0.001]. Education was not a significant predictor of intention change.

## Discussion

This study evaluated an intervention amongst Finnish military conscripts aiming to promote young men’s vegetable consumption by trying to make the prototype of a vegetable eating peer more favorable through a poster campaign. During the 6 months of military service, the three examined prototypes (*Self-regulation, Social status in peer group*, and *Appearance*) changed toward favoring an unhealthy eater with larger changes in the control than in the intervention group. At follow-up, a typical vegetable choosing peer was seen as less self-regulative and a typical peer who avoids vegetables as more self-regulative as well as better looking than at baseline. However, the intervention managed to reduce this negative development, but mainly among those with higher education.

Finnish military service takes place in unique surroundings where young men are surrounded by hundreds of same sex peers. Food choices are made in a social environment that promotes masculinity (Hoikkala et al., 2009), associated with preference for unhealthy foods (Campos et al., 2020). This study showed that military service can indeed exacerbate this problem with a negative effect on vegetable consumption. However, our results indicate that our intervention reduced the decline in vegetable consumption, suggesting some effectiveness of the campaign.

While statistically significant, changes in prototypes were small. However, those prototypes that reacted to the intervention (*Appearance* in the Vegetable Avoider and *Self-regulation* in both Vegetable Chooser and Avoider) were those with the strongest associations with fruit and vegetable consumption as shown in a previous study (Jallinoja et al., 2011) which might explain their having a significant impact on vegetable consumption.

Promoting social images of eaters is novel and thus, plausible effect sizes are unclear. Given the light touch nature of the implemented intervention, the small effect size is, however, not surprising. Future studies trying to intervene through prototype promotion could benefit from incorporating participatory design methods and pre-testing or piloting. For example, a think-aloud study on intervention materials might provide insight into whether the hypothesized processes are evoked as assumed.

To further increase intervention effectiveness, policies easing access to desired behaviors (in this case vegetable consumption) should be implemented (Albarracín et al., 2024). Indeed, another study from the same project found an intervention increasing accessibility of vegetables for conscripts to be more effective than the prototype campaign (Bingham et al., 2012). It is important to remember that the acceptability of such structural campaigns hinges on individual attitudes and norms toward them, so a combination of both would likely be most effective. Such combined interventions could further feature educational campaigns about the benefits of vegetable consumption and support in building healthy eating habits (Ashton et al., 2019).

### Strengths and limitations

The study has good external validity, and a relatively large number of participants of men in an important life phase. However, only half of the participants answered the Vegetable Chooser and Vegetable Abstainer prototypes, limiting statistical power to examine the proposed intervention mechanism of behavior change through prototype change. This was done in order to reduce participant burden and thus risk of lower-quality questionnaire data.

The design is open to criticism concerning conclusions on causality. As the intervention and control arms were not studied simultaneously there is a chance that the effects found in both prototypes and vegetable consumption were not caused by the intervention, but due to unknown time-related changes. However, the adopted design offered logistic benefits and avoided the impact of significant regional differences in vegetable consumption (Helakorpi et al., 2010).

Eating was measured with self-reported food frequency measure instead of more reliable food diaries or recall methods. However, the latter two methods could not have been realistically used in this context with this sample size and food frequency measures have shown adequate validity in earlier studies (Paalanen et al., 2006). As another option, the garrison context would allow for weighing of salad plates to objectively assess how much vegetables were chosen at meals.

It is important to note that data were collected in 2008/2009 and social norms and prototypes around vegetable consumption have undergone noticeable changes since then (Aschemann-Witzel et al., 2021; Faber et al., 2020; McInnes et al., 2023). Also, issues of gender diversity were not as visible in health behavior research as they are today. We therefore cannot claim to have considered diversity of gender or masculinities in the study design. Since then, research has increasingly recognized that there are multiple ways of enacting masculinity, and that food choices may relate differently to these diverse masculinities (Rosenfeld and Tomiyama, 2021). At the same time, recent evidence indicates that, unfortunately, in many contexts masculinity is still associated with “a lot of meat and few vegetables” (Nakagawa and Hart, 2019; Vandello et al., 2024). The military environment, in particular, is one in which masculinity is especially salient (Richard and Molloy, 2020), making it a challenging but also highly relevant context for interventions targeting eating behaviors.

In addition, the media environment during the time of the intervention differed substantially from today. Social media platforms and highly targeted digital marketing were far less prevalent, and the circulation of food-related images operated through different channels. Nevertheless, the basic principle that social images influence eating behavior is likely even more relevant in the current, media-saturated environment, where prototypes of eaters are continually constructed and reinforced through advertising and online culture (Jelicich and Braun, 2023). This suggests that prototype-based approaches may hold even greater potential in contemporary settings.

## Conclusion

This field experiment showed that young men develop more negative prototypes regarding vegetable consumption during their military service but that a light-touch intervention campaign can help buffer these effects to a significant degree. This small but significant effect extended to vegetable consumption which was moderated by participants’ education levels. As such, this study does not support the notion of relying only on light-dose, advert-like interventions. Nevertheless, as cost-effective and easy to disseminate methods, eater prototypes interventions may have potential in promoting public health. It is important to further investigate how to increase their effectiveness.
